# Intraoperative Predictors and Proposal for a Novel Prognostic Risk Score for In-Hospital Mortality after Open Repair of Ruptured Abdominal Aortic Aneurysms (SPARTAN Score)

**DOI:** 10.3390/jcm13051384

**Published:** 2024-02-28

**Authors:** Raffaella Berchiolli, Nicola Troisi, Giulia Bertagna, Mario D’Oria, Luca Mezzetto, Vittorio Malquori, Valerio Artini, Duilio Motta, Lorenzo Grosso, Beatrice Grando, Giovanni Badalamenti, Cristiano Calvagna, Davide Mastrorilli, Gian Franco Veraldi, Daniele Adami, Sandro Lepidi

**Affiliations:** 1 Vascular Surgery Unit, Department of Translational Research and New Technologies in Medicine and Surgery, University of Pisa, 56126 Pisa, Italy; raffaella.berchiolli@unipi.it (R.B.); nicola.troisi@unipi.it (N.T.); giuliaberty.it@hotmail.it (G.B.); vittorio.malquori@outlook.com (V.M.); artini.valerio@gmail.com (V.A.); duilio.motta@outlook.it (D.M.); danieleadami71@gmail.com (D.A.); 2 Vascular and Endovascular Surgery Unit, Cardio-Thoraco-Vascular Department, Azienda Sanitaria Universitaria Giuliano Isontina, 34148 Trieste, Italy; grandobeatrice@gmail.com (B.G.); badalamentigiovanni28@gmail.com (G.B.); calvagnacristiano@gmail.com (C.C.); slepidi@units.it (S.L.); 3 Unit of Vascular Surgery, Department of Cardio-Thoraco-Vascular Surgery, University Hospital and Trust of Verona, University of Verona School of Medicine, 37134 Verona, Italy; luca.mezzetto@aovr.veneto.it (L.M.); lgrosso94@gmail.com (L.G.); mastrodvd87@gmail.com (D.M.); gianfranco.veraldi@aovr.veneto.it (G.F.V.)

**Keywords:** ruptured abdominal aortic aneurysm, open surgical repair, intraoperative scores

## Abstract

(1) Background: Several mortality risk scores have been developed to predict mortality in ruptured abdominal aortic aneurysms (rAAAs), but none focused on intraoperative factors. The aim of this study is to identify intraoperative variables affecting in-hospital mortality after open repair and develop a novel prognostic risk score. (2) Methods: The analysis of a retrospectively maintained dataset identified patients who underwent open repair for rAAA from January 2007 to October 2023 in three Italian tertiary referral centers. Multinomial logistic regression was used to calculate the association between intraoperative variables and perioperative mortality. Independent intraoperative factors were used to create a prognostic score. (3) Results: In total, 316 patients with a mean age of 77.3 (SD ± 8.5) were included. In-hospital mortality rate was 30.7%. Hemoperitoneum (*p* < 0.001), suprarenal clamping (*p* = 0.001), and operation times of >240 min (*p* = 0.008) were negative predictors of perioperative mortality, while the patency of at least one hypogastric artery had a protective role (*p* = 0.008). Numerical values were assigned to each variable based on the respective odds ratio to create a risk stratification for in-hospital mortality. (4) Conclusions: rAAA represents a major cause of mortality. Intraoperative variables are essential to estimate patients’ risk in surgically treated patients. A prognostic risk score based on these factors alone may be useful to predict in-hospital mortality after open repair.

## 1. Introduction

Despite indisputable improvements in both surgical and endovascular techniques and perioperative care, ruptured abdominal aortic aneurysm (rAAA) still represents a life-threatening condition with mortality rates up to 80% [1,2,3]. Mortality outside the hospital remains a main concern, warranting further planning of streamlined transfer networks and vascular surgical departments [4]. Indeed, mortality for rAAA is influenced by multiple factors, such as diagnostic accuracy, efficient transfer to the referral hospital, eligibility for intervention, the presence of well-defined rAAA pathways, and centralization [5,6].

Above all, a fast patient assessment is mandatory to achieve better outcomes. Therefore, accurate preoperative risk stratification could predict who may benefit from surgery, helping both surgeons and patients in making a decision and providing guidance for an appropriate allocation of healthcare resources [7]. In fact, during the first era of open repair, several risk scores were developed in an attempt to predict mortality after open surgical repair (OSR), including the Glasgow Aneurysm Score (GAS), the Vancouver scoring system, the Edinburgh Ruptured Aneurysm Score, the Hardman Index, and the RAAA Physiological and Operative Severity Score for the enUmeration of Mortality and Morbidity (RAA-POSSUM) [8,9,10,11,12]. More recently, in the first era of endovascular aneurysm repair (EVAR), the Vascular Study Group of New England (VSGNE) Ruptured Aneurysm Score, the Harborview Medical Center (HMC) score, and the Dutch Aneurysm Score (DAS) were developed for the same purpose [13,14,15]. These predictive scores only included preoperative variables and presented several ceilings, thereby limiting their validation among the general population. Indeed, because of the low positive predictive value for death and major morbidity, both GAS and Hardman indices seem to be of limited value in clinical decision making, especially in high-risk patients [16,17]. Similarly, the RAA-POSSUM has fallen out of favor not only due to its calculation difficulty but also because it overestimates the number of patients at risk of death. An exception to the previous models was VSGNE, which was initially developed on an open repair patient population and was the only one including suprarenal aortic clamping. Overall, the main limitation of all described risk scores is their purpose—to support the decision to deny surgical intervention in high-risk patients. On the other hand, predicting a low risk of mortality often leads to offering treatment to patients who are otherwise turned down for surgery. Moreover, considering the improvements in anesthesiologic management and perioperative care, an increasing number of rAAA patients are offered treatment. In this context, the index should be used not to deny an operation but to predict the outcome after surgery. In the current literature, there is a lack of studies specifically focused on intraoperative factors affecting mortality. Moreover, an index that considers only intraoperative variables has never been described.

The aim of the present study was therefore to identify intraoperative factors affecting early outcomes after open repair for rAAA and develop and internally validate a novel prognostic risk score for in-hoSPital mortality after the open repair of ruptured abdominal aortic aneurysms (SPARTAN score).

## 2. Materials and Methods

### 2.1. Study Design

Between January 2007 and October 2023, 511 patients were treated for non-ruptured or ruptured aorto-iliac aneurysms in three tertiary referral centers.

Only patients with a diagnosis of rupture observed in computed tomography angiography (CTA) scans and undergoing open surgical repair (OSR) were included in the present study.

The exclusion criteria were as follows:

- Thoracoabdominal/suprarenal/pararenal aortic aneurysms;
- Symptomatic patients without CT signs of rupture;
- Isolated iliac aneurysms;
- Previous open/endovascular AAA repair;
- Death during operation time.

All data were retrospectively extracted from the hospital’s medical records, and 316 patients were finally included in the present series (246 Pisa, 41 Verona, 29 Trieste). Pisa University Hospital collects a total volume of 20 cases of rAAA per year, whereas Verona and Trieste collect a noticeably smaller number of 10 and 8 per year, respectively. All these centers can provide at least two surgeons with a minimum of ten years of experience who are able to equally perform open and endovascular surgery such that the treatment can be customized with respect to a single patient. Moreover, the presence of a similar aortic team at each center allows the minimization of the difference between the care provided.

The three participating centers serve similar-sized populations in three different Italian regions. Local institutional protocols were in place at each center for the care of acute aortic diseases.

All patients underwent CTA with at least 1.5 mm reconstruction of the entire aorta before the procedure.

Other definitions of clinical events and/or imaging parameters were in accordance with the reporting standards of the Society for Vascular Surgery [18].

Institutional Review Board approval was waived due to the retrospective nature of the study, and all patients gave their written consent to the procedure approved by the Ethics Committee.

### 2.2. Statistical Analysis

Continuous data were expressed as the mean ± standard deviation (SD) or median ± interquartile range (IQR) values when necessary. Categoric data were expressed as percentages. The nonparametric Pearson chi-square test was used when necessary to compare variables.

Statistical significance was defined at the *p* < 0.05 level. The intra-operative factors analyzed were as follows: presence of hemoperitoneum (intraoperatively detected), operation time, type of clamping (suprarenal, infrarenal, or supraceliac), type of aortic reconstruction (tube graft, aorto-biiliac or bifemoral graft, and aorto-bifemoral graft), patency of at least one hypogastric artery, inflammatory aneurysm, and mycotic aneurysm.

Univariate analyses of intraoperative factors affecting in-hospital mortality were performed by means of a log-rank test. Associations of intraoperative variables with in-hospital mortality rates were sought based on a multinomial logistic regression in order to identify the independent predictors of in-hospital mortality rates. Cox regression was applied to all variables, with a *p* level of <0.05, selected during the univariate analysis. Independent variables were selected via a stepwise method. Potentially confounding factors, such as age, sex, frailty, and cardiovascular risk profile, were considered to increase the robustness of statistical analyses. Therefore, a stratification into different layers according to potential predictors was performed to adjust for confounding factors. The results of the regression model were presented as the odds ratio (OR) and 95% CI.

Independent intraoperative predictors (positive and negative) were used to create a prognostic score for in-hospital mortality. In order to calculate the score in a simplified way, a numerical value of +/−1 was assigned for an approximate OR value of 3. The risk stratification for in-hospital mortality was carried out on the basis of the score values in order to define three subgroups: low, moderate, and high.

Statistical analysis was performed using SPSS software (version 24.0 for Apple; IBM Corporation, Armonk, NY, USA).

## 3. Results

### 3.1. Study Population

After the application of inclusion/exclusion criteria, mentioned in the Section 2 (excluding thoracoabdominal aortic aneurysms, pararenal aortic aneurysms, only symptomatic patients, thrombus fissuration, isolated ruptured hypogastric aneurysms, previous open or endovascular abdominal aortic repair, and death during operation time), data analysis was performed on 316 patients. Demographics and preoperative data are reported in Table 1.

The mean operation time was 218.6 ± 80 min. With regard to clamping, the site was suprarenal (140 cases, 44.9%), infrarenal (132 cases, 41.2%), or supraceliac (44 cases, 13.9%). A tube graft was performed in 165 cases (52.2%), whilst in 135 cases (42.7%), an aorto-bi-iliac graft was made; in the remaining 6 cases (5.1%), an aorto-bi-femoral graft was performed.

With regard to aneurysm morphology, 17 patients (5.4%) had an inflammatory aneurysm. In seven cases (2.2%), a mycotic aneurysm was diagnosed.

Overall, the in-hospital mortality rate was 30.7% (97 cases). The causes of death are as follows: cardiac (heart failure and fatal arrhythmias) in 28 patients (28.9%), bleeding complications in 20 patients (20.6%), respiratory complications (pneumonia) in 6 patients (6.2%), bowel ischemia in 7 patients (7.2%), and multiorgan failure (MOF) in 36 cases (37.1%).

### 3.2. Intraoperative Factors: Univariate Analysis

The univariate analysis showed that hemoperitoneum was the main intraoperative predictor of in-hospital mortality. Suprarenal clamping and operation times of >240 min also affected the in-hospital mortality rate. Conversely, the patency of at least one hypogastric artery seemed to have a protective role.

Table 2 shows a univariate analysis of intraoperative factors affecting in-hospital mortality.

### 3.3. Prognostic Score for In-Hospital Mortality after Open Repair of Ruptured Abdominal Aortic Aneurysms (SPARTAN)

On the basis of the odds ratio obtained with multinomial logistic regression, the following numerical values could be assigned to the following parameters: +4 hemoperitoneum (OR 13.788), +1 operation time >240 min (OR 4.381), and +2 suprarenal clamping (OR 6.187). Furthermore, the patency of at least one hypogastric artery (protective factor) could receive a numerical value of −2 (OR 6.450).

Specifically, each increase in an odds ratio of 3 corresponds to a 1-point increase in our score. For the protective factor—the patency of at least one hypogastric artery—we used the same principle. We standardized this value in order to obtain a single cipher score, which is easily and rapidly calculable. In the case of a decimal number in the OR, we rounded the cipher. Furthermore, based on the Exp(B), we confirmed the score attributed to each single parameter.

Table 3 shows a multinomial logistic regression analysis of intraoperative factors affecting in-hospital mortality.

On the basis of this distribution, the risk stratification for in-hospital mortality was carried out by merging the patients with a score value ranging from −2 to 0 (low risk), from 1 to 4 (moderate risk), and from 5 to 7 (high risk).

In the present population study, 155 patients (49%) could be identified as low risk, 122 patients (38.6%) as moderate risk, and 39 patients (12.4%) as high risk. The in-hospital mortality rate was 15.5% for low-risk patients (24 deaths), 39.3% for moderate-risk patients (48 deaths), and 64.1% for high-risk patients (25 deaths).

**Figure 1:**
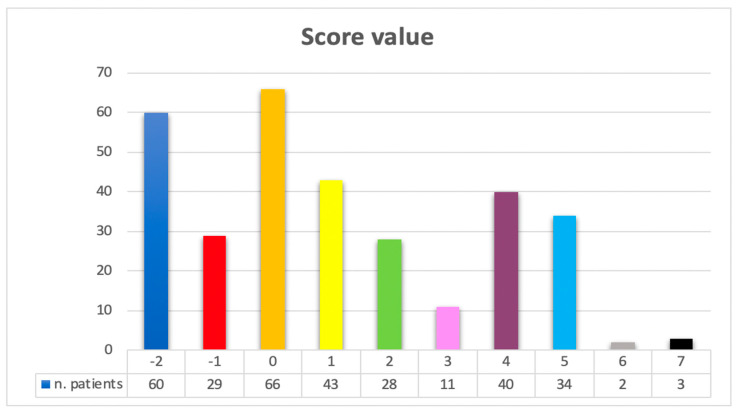
Distribution of patients according to the score values.

Figure 1 shows the distribution of the patients according to the score values.

## 4. Discussion

This study demonstrates that the SPARTAN score accurately stratifies surgically treated patients in different risk categories for in-hospital mortality, based on only four intraoperative factors. With regard to univariate analysis, independent negative predictors of perioperative mortality were hemoperitoneum, suprarenal clamping, and operation times of >240 min, while the patency of at least one hypogastric artery seemed to have a protective role. Based on the multinomial logistic regression, each one of these parameters was given a whole numerical value according to the respective odds ratio, thereby creating an intuitive and easy-to-calculate prognostic tool. Differently from what was previously described, we developed a score specifically for surgically treated patients, considering only intraoperative variables instead of preoperative ones. Furthermore, based on this combination of parameters, we calculated the relative mortality and stratified patients accordingly. This score allows us to classify patients who underwent OSR as low, intermediate, and high-risk.

Indeed, almost all previous mortality risk scores focused on the identification of preoperative factors possibly affecting mortality in rAAA patients to predict perioperative outcomes after surgery, be it open or endovascular. Moreover, several studies concentrated on the identification of preoperative parameters influencing long-term survival [18,19,20]. Prediction models were mainly developed to aid the decision regarding whether lifesaving treatment should be withheld. However, a recent study comparing different predictive models demonstrated a comparable performance, although an almost perfect prediction would be necessary to withhold intervention, and no existing scoring system is capable of that [21,22]. Among these preoperative scores, VSGNE was the only one that included suprarenal aortic clamping in its formula, witnessing the pivotal role played by intraoperative factors in determining early mortality. In this scenario, we created an intuitive index, which may predict in-hospital mortality after OSR, assuming that all rAAA patients were offered treatment. This latter concept is representative of the Italian mindset that is common among the three centers involved, i.e., the tendency to guarantee a chance of treatment anyhow. As described in our previous analysis, here, we confirm the role of hemoperitoneum, suprarenal clamping, and operation times of >240 min as factors independently affecting early mortality [23,24]. These findings are in line with those reported in the current literature. Indeed, several studies underlined that total blood loss and intraoperative hypotension are significant predictors of death [25,26,27,28]. In our analysis, we considered hemoperitoneum as an indirect and non-numeric measure of blood loss, but it is intraoperatively objectifiable. The latter is strictly linked to hypotension, which worsens surgical outcomes. Surely, factors such as age, sex, patient’s frailty, and cardiovascular status play a pivotal role, and they should be considered as potential confounders that could have influenced the results. In any case, the presence of hemoperitoneum may be used as a surrogate of a precise quantification of hemorrhage, which exemplifies the purpose of our score—roughly calculates mortality through identifiable intraoperative findings. Indeed, hemoperitoneum is the worst parameter affecting mortality in our study. As a further confirmation of this finding, Kim et al. [28] showed that the operative control of bleeding, maintenance of systemic perfusion, and infrarenal aortic clamping in infrarenal rAAA, regardless of neck length, as possible means to avoid additional renal ischemic changes are vital. Obviously, suprarenal aortic clamping in juxtarenal or suprarenal rAAA is obligatory for anatomical reasons. In any case, intraoperative bleeding control is one of the main predictors of perioperative death during OSR [29,30,31,32].

Moreover, the first attempt to focus only on intraoperative variables affecting mortality after open repair was carried out by Davidović et al. [29], even though a proper mortality score based on these parameters has never been developed. The study showed that intraoperative determinants of increased mortality were an aortic cross-clamping time of >47 min, duration of surgery of >200 min, intraoperative blood loss of >3500 mL, diuresis of <400 mL, arterial systolic pressure of <97.5 mmHg, and the need for aorto-bifemoral bypass. Similarly, in our analysis, an operative time of longer than 240 min is a negative predictor of mortality. Indeed, the duration of surgery is directly linked to prolonged aortic clamping and complex arterial reconstructions, increasing blood loss. It has already been demonstrated that prolonged (>50 min) aortic clamping is an independent predictor of postoperative renal and cardiac dysfunction, even in elective surgery [33]. Moreover, patients with longer operative times and increasing aneurysm diameter have an increased risk of renal damage [34]. On the other hand, reducing operative time is not always feasible, especially in case of severe coagulopathy. Acidosis is, besides hypothermia and hemodilution, a possible cause of coagulopathy, which often occurs in rAAA [35]. Therefore, more time is spent on performing hemostasis, rather than achieving effective clamping. Indeed, intense anesthesiologic management that is able to correct hemodynamic instability, acidosis, and electrolytic balance is fundamental.

Furthermore, in this scenario, the protective role of hypogastric artery patency cannot be neglected. During both OSR and EVAR, all attempts should be made to preserve pelvic perfusion by maintaining antegrade flow to at least one patent internal iliac artery in order to avoid all potential complications due to its coverage [36,37]. Recent investigations showed that concomitant hypogastric artery embolization with EVAR is associated with longer and more complicated hospital stays. Ischemic colitis is a rare complication of EVAR; however, hypogastric embolization increases the risk of ischemic colitis and renal failure requiring dialysis [38]. Furthermore, the number of hypogastric arteries is crucial for safe outcomes after AAA repair. The main variables related to death were bowel ischemia (BI), emergency aortoiliac repair, and the number of internal iliac arteries (IIA) preserved. Above all, the main factors related to BI were the number of IIAs in postoperative emergency aortoiliac repair and cardiac diseases [39].

Although the aforementioned studies analyzed preoperative and intraoperative factors affecting outcomes after open surgery for rAAA, a prognostic score with only intraoperative variables has never been developed. With our score, we aim to promptly classify patients according to a few objectifiable intraoperative findings. The majority of patients in our cohort were classified as low risk regarding in-hospital mortality, with a score between −2 and 0 and an early mortality of 15.5%. On the other hand, moderate and high-risk patients experienced a mortality rate of 39.3% and 64.1%, respectively. This risk stratification seems to linearly and effectively correlate with mortality rates. In fact, as the score increases, mortality increases accordingly, doubling the risk when moving from one category to the following. In this case, a properly obtained risk score represents a useful tool that informs surgeons about what the outcomes after open repair might be and facilitates communication with relatives about the prognosis. Obviously, the SPARTAN score alone, as all other proposed scores, is not one hundred percent reliable. Indeed, it does not consider potential complications that may occur during surgery and neither the patient’s preoperative conditions nor the surgeon’s skills. Furthermore, it has been settled on patients undergoing OSR, excluding a priori those who underwent EVAR. The decision to customize a score only on surgically treated patients derives from the observation that a routine practice of OSR appears to give better results than EVAR despite what is described in several trials [40,41]. In addition, our study population belongs to high-volume centers with great expertise in rAAA management, where the advantages of EVAR are marginal and partly offset by the surgeons’ ability in open surgery, even in more complex cases that are otherwise endovascularly untreatable. However, the primary objective of this study is to provide surgeons with an adjunctive tool that is capable of estimating early mortality and not to support the superiority of one treatment over another. In fact, the choice between EVAR and OSR must be tailored to a single patient, considering the whole preoperative assessment. Hypothetically, the SPARTAN score may be used, in addition to other validated preoperative scores, to give extra numerical information about rAAA the patients’ prognosis. Once the risk of death is calculated based on preoperative factors included in predictive models, surgeons may selectively apply our score to calculate the risk of in-hospital mortality after open repair.

### Study Limitations

This study has some limitations. The primary limit is the retrospective nature of data collection, which is particularly challenging in the setting of emergency surgery and could result in a small proportion of missing data. Then, the relatively small sample size reduces the sensitivity of multivariate analyses and the generalizability of results. Moreover, our index has only been internally validated, so external validation is required to confirm our findings. The surgeons’ ability is also a pivotal factor that may influence outcomes, especially in emergently treated patients; in this sense, the sole variable that can be part of a calculated score might be the surgeon’s volume, which is a well-established factor affecting outcomes. However, this was beyond the purpose of our score, which mainly aimed to quantify the weight of the identified intraoperative variables rather than preoperative factors.

## 5. Conclusions

RAAA still represents a life-threatening condition with high perioperative mortality rates, and this is also observed in the contemporary endovascular era. When endovascular repair is not feasible, open repair remains a life-saving alternative, and several intraoperative factors may play a pivotal role in determining postoperative outcomes. Hemoperitoneum, suprarenal aortic clamping, and operation times of >240 min were negative predictors of in-hospital mortality, whereas the patency of at least one hypogastric artery had a protective role. The prognostic SPARTAN risk score, based on these factors alone, may allow surgeons to predict in-hospital mortality in patients undergoing open repair for rAAA. Furthermore, it may be useful to stratify treated patients into three different categories—low, intermediate, and high risk—and improve communication between surgeons and relatives regarding prognoses. However, further studies in a large prospective series will be necessary to externally validate this score.

## Figures and Tables

**Table 1:** Demographics and preoperative data.

Demographic Features	*n*. Patients = 316
Age (mean ± SD)	77.3 ± 8.5
Age > 80 years	131 (41.5%)
Male sex	208 (65.8%)
Comorbidities	
- Hypertension	237 (75%)
- Coronary artery disease	78 (24.7%)
- Atrial fibrillation	42 (13.3%)
- Chronic obstructive pulmonary disease	94 (29.7%)
- Diabetes mellitus	236 (11.4%)
- Chronic kidney disease	63 (19.9%)
- History of malignancy	24 (7.6%)
- Previous abdominal surgery	32 (10.1%)
Preoperative clinical status	
- Resuscitation maneuvers	43 (13.6%)
- Loss of consciousness	104 (32.9%)
- Endotracheal intubation before surgery	68 (21.5%)
- Blood transfusions	115 (36.4%)
- Hemorrhagic shock	152 (48.1%)

Continuous data are presented as the means; categorical data are given as the counts (percentage). SD, standard deviation.

**Table 2:** Univariate analysis of intraoperative factors affecting in-hospital mortality.

Variables	*n*. Patients Alive (219)	*n*. Patients Dead (97)	*p*
Hemoperitoneum			
- yes	61 (27.9%)	51 (52.6%)	**<0.001**
Operation time > 240 min			
- yes	73 (33.3%)	47 (48.5%)	**0.008**
Suprarenal clamping			
- yes	115 (52.5%)	69 (71.1%)	**0.001**
Supraceliac clamping			
- yes	27 (12.3%)	17 (17.5%)	0.146
Tube graft			
- yes	106 (48.4%)	45 (46.4%)	0.957
Patency of at least one hypogastric artery			
- yes	210 (95.9%)	85 (87.6%)	**0.008**
Inflammatory aneurysm			
- yes	15 (6.8%)	2 (2.1%)	0.132
Mycotic aneurysm			
- yes	6 (2.7%)	1 (1%)	0.094

Bold identifies statistically significant *p* values (i.e., *p* value <0.05).

**Table 3:** Multinomial logistic regression analysis of intraoperative factors affecting in-hospital mortality.

Variables	B	Standard Error	Odds Ratio	*p*	Exp(B)	95% CI for Exp(B)	
Hemoperitoneum	0.973	0.262	13.788	**<0.001**	2.645	1.583	4.420
Operation time > 240 min	0.553	0.264	4.381	**0.036**	1.738	1.036	2.917
Suprarenal clamping	0.689	0.277	6.187	**0.013**	1.991	1.157	3.426
Patency of at least one hypogastric artery	−1.236	0.487	6.450	**0.011**	0.290	0.112	0.754

CI, confident interval. Bold identifies statistically significant *p* values (i.e., *p* value < 0.05).

## Data Availability

The data presented in this study are available on request from the corresponding author. The data are not publicly available due to privacy.
